# Efficacy of orthokeratology combined with atropine versus orthokeratology alone for myopia control in children: a meta-analysis

**DOI:** 10.3389/fmed.2026.1786718

**Published:** 2026-07-24

**Authors:** Yuchan Li, Meichao Yi, Bing Liu

**Affiliations:** Department of Ophthalmology, Zigong Fourth People's Hospital, Zigong, Sichuan, China

**Keywords:** atropine, children, meta-analysis, myopia control, orthokeratology

## Abstract

**Objective:**

To systematically evaluate the efficacy of orthokeratology combined with atropine versus orthokeratology alone in treating myopia in children.

**Methods:**

Randomized controlled trials (RCTs) comparing the efficacy of orthokeratology combined with atropine versus orthokeratology alone for pediatric myopia were retrieved from databases including CNKI, Wanfang, VIP, CBM, PubMed, Cochrane Library, Embase, and Web of Science from inception to April 2025. Data were analyzed using RevMan 5.2 software.

**Results:**

A total of 18 randomized controlled trials involving 1,669 myopic children were included. The analysis showed that the intervention group had greater improvements in axial elongation (SMD = −0.66, 95% CI = −0.80 to −0.53, *p* < 0.001), tear film break-up time (SMD = 0.48, 95% CI = 0.33 to 0.63, *p* < 0.001), and corneal curvature (SMD = −2.47, 95% CI = −4.21 to −0.73, *p* = 0.005) compared to the control group. However, no statistically significant differences were found between the two groups in uncorrected visual acuity (SMD = 0.49, 95% CI = −0.27 to 1.24, *p* = 0.21) or tear film lipid layer thickness (SMD = −0.00, 95% CI = −1.09 to 1.09, *p* = 1.00). In addition, increased pupil diameter (SMD = 0.37, 95% CI = 0.09 to 0.64, *p* < 0.001) and reduced accommodative amplitude (SMD = −1.50, 95% CI = −2.58 to −0.43, *p* = 0.006) reflected the side effects associated with combined atropine treatment.

**Conclusion:**

Compared with orthokeratology alone, orthokeratology combined with atropine treatment reduces axial elongation, tear film break-up time, and corneal curvature in myopic children, but shows no significant advantage in uncorrected visual acuity or tear film lipid layer thickness, while incurring side effects of increased pupil diameter and reduced accommodative amplitude.

## Introduction

Myopia has become a critical public health issue worldwide, with a particularly rapid increase among children and adolescents. Epidemiological studies indicate that the prevalence of myopia in East Asian children aged 10–15 years has exceeded 70%, with approximately 20% progressing to high myopia, significantly elevating the risk of irreversible vision impairment (1, 2). The cornerstone of myopia control lies in slowing axial elongation. Currently, orthokeratology and low-dose atropine are two mainstream interventions, exerting therapeutic effects through optical reshaping and pharmacological modulation, respectively (3, 4). Orthokeratology, worn overnight, utilizes reverse geometry mechanics to effectively reduce peripheral retinal hyperopic defocus. As a standalone treatment, its efficacy in myopia control is well-established; a comprehensive review indicates that orthokeratology can inhibit axial elongation by 32 to 63% over two years compared to single-vision lenses (5). Meanwhile, low-dose atropine is believed to modulate retinal and scleral pathways, operating through a distinct pharmacological mechanism (6). Given their complementary modes of action, combining orthokeratology with atropine presents a promising strategy to maximize myopia control efficacy, a premise supported by initial clinical studies (6). Recent clinical studies suggest that combination therapy may yield synergistic effects, demonstrating superior outcomes compared to monotherapy (7, 8). However, existing research exhibits significant heterogeneity in treatment protocols, efficacy evaluation metrics, and follow-up durations. Systematic assessments are still needed for visual quality, changes in accommodative amplitude, and corneal biomechanical properties under combination therapy. This meta-analysis aims to systematically evaluate the efficacy of orthokeratology combined with atropine versus orthokeratology monotherapy in pediatric myopia control. The findings will provide evidence-based guidance for individualized treatment strategies in clinical practice and propose recommendations for future research directions.

## Methods

### Inclusion and exclusion criteria

#### Inclusion criteria

(1) Study type: Randomized controlled trials (RCTs), with no restrictions on blinding. Publications were limited to those in Chinese or English.
(2) Participants: Individuals aged <18 years diagnosed with myopia.
(3) Interventions: Participants in the two groups were treated with either orthokeratology combined with atropine or orthokeratology alone.
(4) Outcome measures: Axial elongation; Uncorrected visual acuity; Tear break-up time; Tear film lipid layer thickness; Pupil diameter; Accommodative amplitude; Corneal curvature. Included studies had to report at least one of the above outcomes.

#### Exclusion criteria

(1) Studies with insufficient data, duplicate publications, or unavailable full texts.
(2) Non-RCTs, reviews, conference abstracts, case reports, or animal studies.

### Search strategy

Computerized searches were conducted in the following databases: CNKI, Wanfang, VIP, China Biology Medicine (CBM), PubMed, Cochrane Library, Embase, and Web of Science. The search period spanned from inception to April 2025. The search terms included: “Atropine,” “Orthokeratology” OR “Orthokeratology lenses,” “Myopia” OR “Myopia control,” “Child” OR “Children.” A combination of Medical Subject Headings (MeSH) terms and free-text words was employed. Additionally, the reference lists of included studies were manually screened. The PubMed search strategy is provided as an example: ((((child[MeSH Terms]) OR (children[Title/Abstract])) AND ((Myopia[MeSH Terms]) OR (myopia control[Title/Abstract]))) AND (atropine[MeSH Terms])) AND ((orthokeratology[Title/Abstract]) OR (orthokeratology lenses[Title/Abstract])). As far as applicable, the review was conducted according to the PRISMA guideline.

### Literature screening and data extraction

The literature screening process initially employed the NoteExpress computerized literature management system for automatic duplicate checking. After deduplication, preliminary screening was conducted by reviewing titles and abstracts. Subsequently, full texts of the remaining articles were evaluated to exclude those that did not meet the inclusion criteria. For data extraction, two researchers independently performed the extraction based on the predefined inclusion and exclusion criteria, following a blinded approach. In cases of disagreement between the two researchers, a third researcher was consulted to assist in resolving discrepancies. The extracted data included: title, author(s), publication date, age (of participants), sample size, intervention measures, drug dosage, treatment duration, and outcome indicators.

### Quality assessment of literature

Two researchers independently evaluated the quality of the included literature according to the criteria outlined in the Cochrane Handbook for Systematic Reviews of Interventions (Version 5.1.0) (9). The following aspects were evaluated: whether the study describes the specific method and process of random sequence generation; whether the study describes the method of allocation concealment; whether the study prevents all participants and intervention providers from accessing intervention information; whether the study provides assessors with effective intervention information; whether outcome data are completely reported for each study, including any missing data; whether the study selectively reports effective or ineffective outcomes; and other biases. Studies that fully meet the above criteria are rated as Grade A; those that partially meet them are rated as Grade B; and those that do not meet them at all are rated as Grade C. If the evaluations by two researchers are inconsistent, a third researcher will be consulted to reach a final determination.

### Statistical methods

The analysis was performed using RevMan 5.2 statistical software, with the I^2^ test applied to assess clinical heterogeneity. When *p* ≥ 0.05 and I^2^ < 50%, it indicates that there is no statistical heterogeneity among the studies, and a fixed-effects model is used for meta-analysis. Conversely, when these criteria are not met, it suggests the presence of statistical heterogeneity among the studies. In such cases, the source of the differences should first be analyzed, and sensitivity analysis should be performed by excluding studies one by one to explore the source of heterogeneity. If there is no significant clinical heterogeneity and no definite source of statistical difference can be identified, a random-effects model is adopted for meta-analysis. All outcome measures included in this study were continuous variables; thus, the standardized mean difference (SMD) with its 95% confidence interval (CI) was used as the effect size. A *p*-value < 0.05 was considered statistically significant. For indicators with seven or more included studies, publication bias was assessed by visual inspection of funnel plots combined with Egger’s test. If publication bias existed, the trim-and-fill method was used to explore the potential impact of unpublished studies on the results.

## Results

### Basic characteristics and quality assessment of included studies

**Figure 1:**
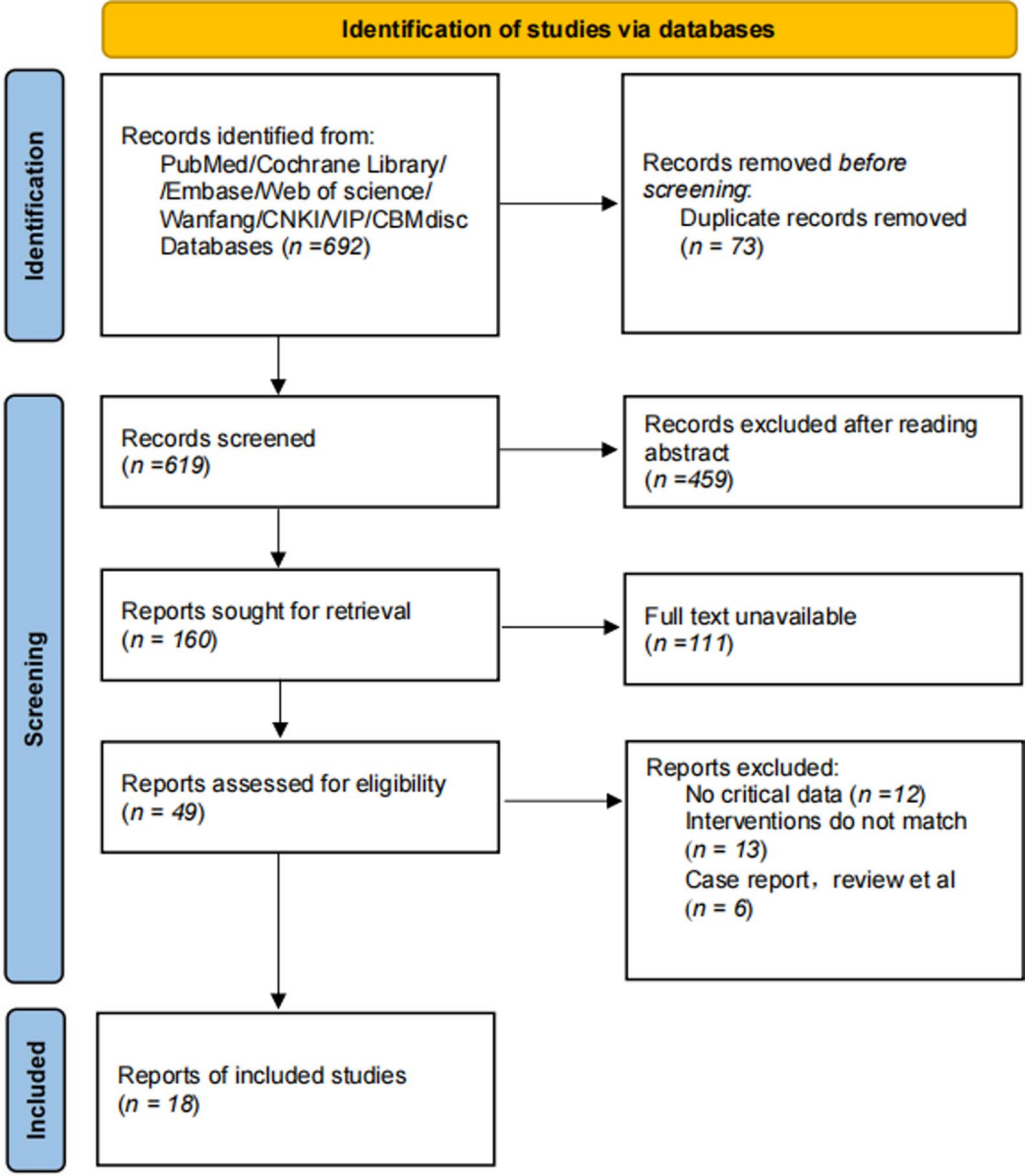
Literature screening flowchart.

A total of 692 articles were retrieved from the database search, and 18 studies (10–27) were ultimately included. The literature screening process is shown in Figure 1.

**Figure 2:**
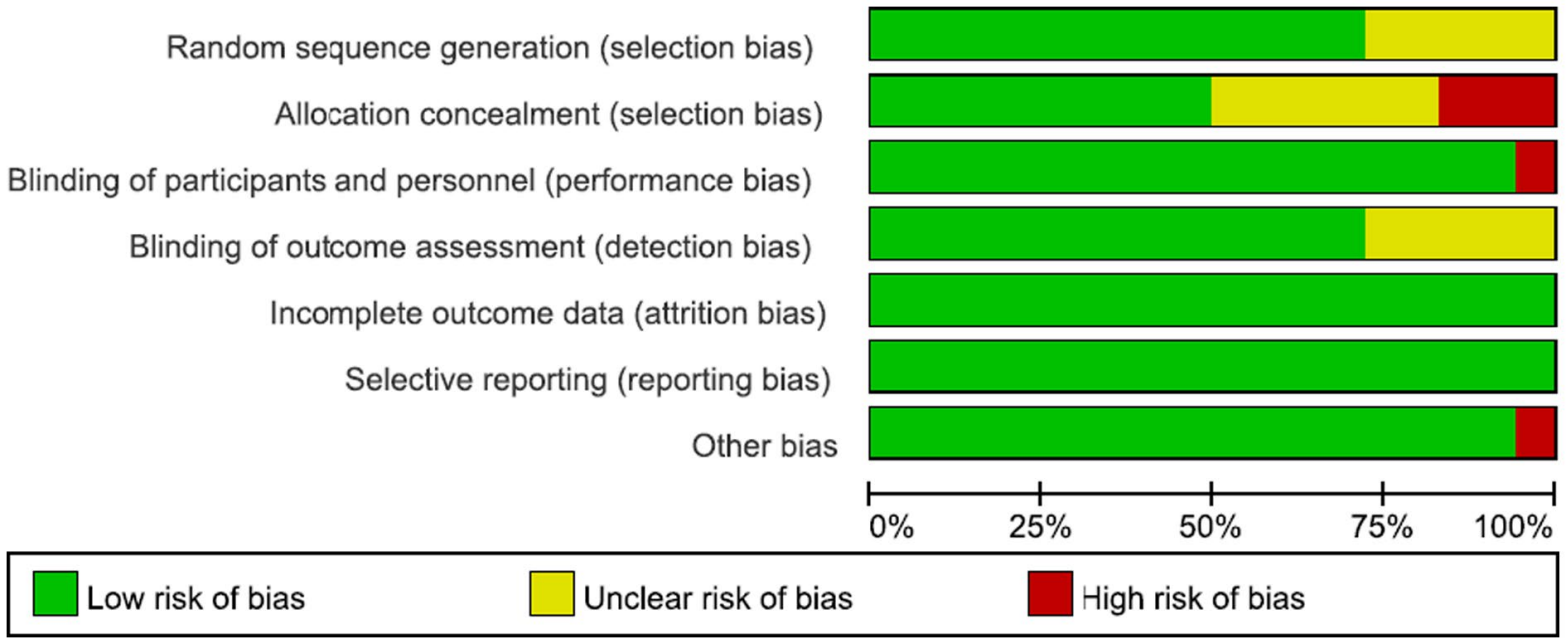
Risk of bias assessment graph for included literature.

The included studies comprising 9 English-language articles and 9 Chinese-language articles. These studies involved 1,669 myopic children, with 834 cases in the orthokeratology combined with atropine group and 835 cases in the orthokeratology-alone group. The basic characteristics of the included studies are presented in Table 1. Quality assessment of the included studies revealed 7 Grade A articles and 11 Grade B articles. The risk of bias assessment graph for the included studies is shown in Figure 2.

**Table 1:** Basic characteristics of included literature.

Author and year	Country	Sample Size		Age		Intervention group	Control group	Follow-up period	Assessment tool
		Control group	Intervention group	Intervention group	Control group				
Wan, 2024 (10)	China	50	50	10.92 ± 1.20	10.73 ± 1.23	Orthokeratology+0.01%Atropine Eye Drops	Orthokeratology + Placebo Eye Drops	1 month	③④⑤⑥
Li, 2023 (11)	China	45	45	13.90 ± 2.40	13.20 ± 2.80	Orthokeratology+0.01%Atropine Eye Drops	Orthokeratology	12 months	③⑦
Yan, 2022 (12)	China	30	30	11.27 ± 1.14	11.34 ± 1.16	Orthokeratology+0.01%Atropine Eye Drops	Orthokeratology	12 months	②③④⑦
Zhang, 2021 (13)	China	41	41	-	-	Orthokeratology+0.01%Atropine Eye Drops	Orthokeratology	12 months	①③④⑦
Cheng, 2021 (14)	China	52	53	13.42 ± 1.01	13.98 ± 1.10	Orthokeratology+0.01%Atropine Eye Drops	Orthokeratology	6 months	②⑤⑥
Huang, 2021 (15)	China	50	50	12.20 ± 0.30	12.30 ± 0.30	Orthokeratology+1%Atropine Eye Drops	Orthokeratology	12 months	②
Liu, 2021 (16)	China	49	49	12.11 ± 1.04	12.03 ± 1.07	Orthokeratology+0.01%Atropine Eye Drops	Orthokeratology	12 months	②③④⑦
Li, 2019 (17)	China	20	20	13.60 ± 3.78	13.25 ± 3.70	Orthokeratology+0.01%Atropine Eye Drops	Orthokeratology	12 months	①
Gao, 2021 (18)	China	70	70	10.50 ± 2.00	11.00 ± 2.00	Orthokeratology+0.01%Atropine Eye Drops	Orthokeratology	12 months	③④
Vincent, 2020 (19)	Australia	28	25	9.10 ± 1.10	8.90 ± 1.20	Orthokeratology+0.01%Atropine Eye Drops	Orthokeratology	6 months	①⑤
Tan, 2019 (20)	China	34	30	9.09 ± 1.11	9.09 ± 1.17	Orthokeratology+0.01%Atropine Eye Drops	Orthokeratology	1 months	①②
Yu, 2022 (21)	China	26	27	9.80 ± 1.64	10.07 ± 1.44	Orthokeratology+0.01%Atropine Eye Drops	Orthokeratology + Placebo Eye Drops	12 months	①⑤⑥
Xiao, 2023 (22)	China	170	170	10.78 ± 1.23	10.81 ± 1.34	Orthokeratology+0.01%Atropine Eye Drops	Orthokeratology	12 months	①③
Kinoshita, 2020 (23)	Japan	35	38	10.37 ± 1.65	10.33 ± 1.59	Orthokeratology+0.01%Atropine Eye Drops	Orthokeratology	24 months	①②
Tan, 2023 (24)	China	35	34	9.10 ± 1.20	9.20 ± 1.00	Orthokeratology+0.01%Atropine Eye Drops	Orthokeratology	24 months	①⑥
Zhao, 2021 (25)	China	36	39	10.33 ± 1.65	10.23 ± 1.11	Orthokeratology+0.01%Atropine Eye Drops	Orthokeratology	1 month	①
Hao, 2021 (26)	China	24	21	10.13 ± 1.19	10.10 ± 1.22	Orthokeratology+0.01%Atropine Eye Drops	Orthokeratology	12 months	①
Xu, 2023 (27)	China	40	42	-	-	Orthokeratology+0.01%Atropine Eye Drops	Orthokeratology + Placebo Eye Drops	24 months	③④

① Axial elongation; ② Uncorrected visual acuity (UCVA); ③ Tear break-up time (TBUT); ④ Tear film lipid layer thickness (LLT); ⑤ Pupil diameter; ⑥ Accommodative amplitude; ⑦ Corneal curvature.

### Meta-analysis results

#### Axial elongation

**Figure 3:**
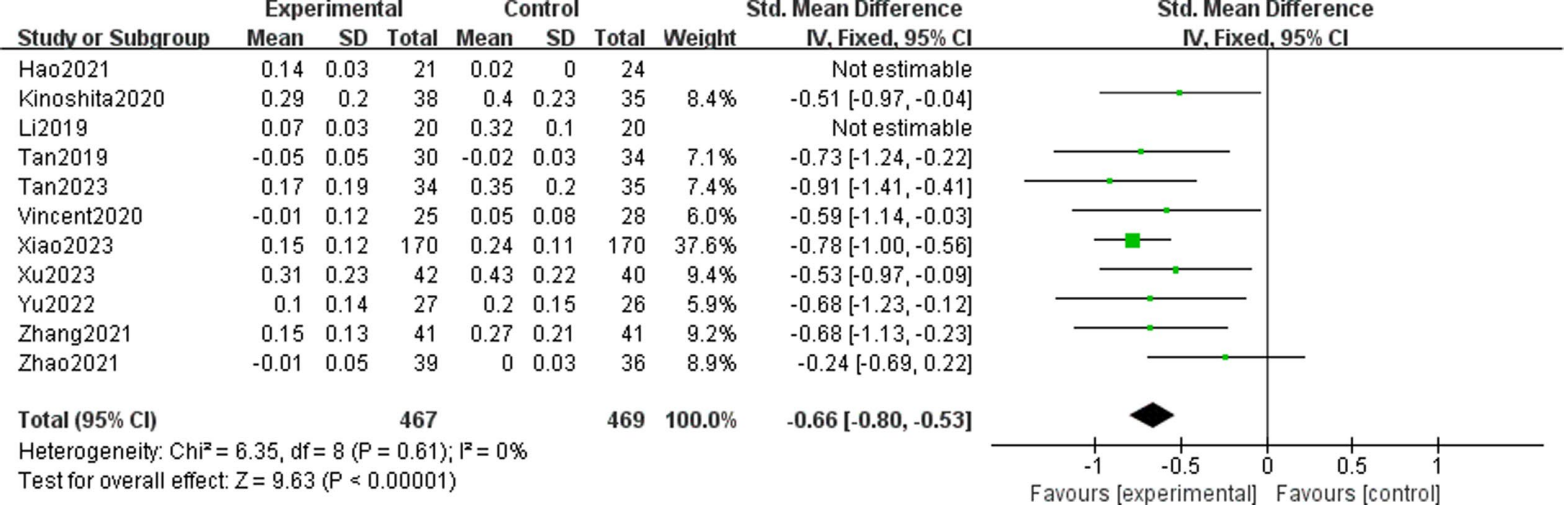
Forest plot of the meta-analysis for axial elongation.

**Figure 4:**
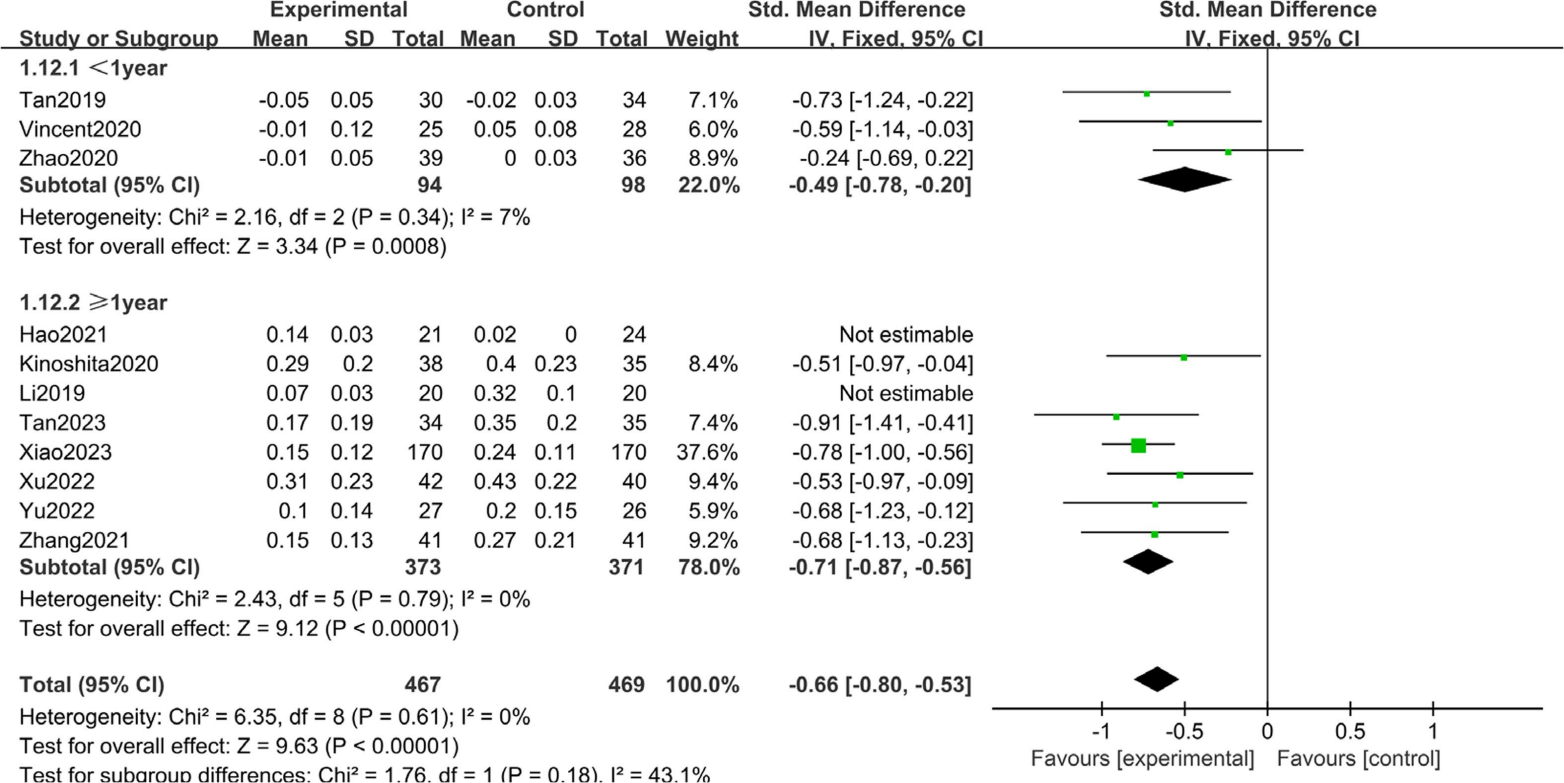
Forest plot of subgroup analysis by follow-up time for axial elongation.

A total of 11 studies (13, 17, 19–27) reported axial elongation, with significant heterogeneity among the studies (*p* < 0.001, I^2^ = 78%). After sensitivity analysis excluding the study by Li et al. (17), no heterogeneity was observed (*p* = 0.61, I^2^ = 0%), and thus a fixed-effects model was adopted. The meta-analysis demonstrated that the axial elongation in the intervention group was significantly lower than that in the control group (SMD = −0.66, 95% CI = −0.80 to −0.53, *p* < 0.001), as shown in Figure 3. Subgroup analysis based on follow-up duration indicated that axial elongation in the intervention group was lower than that in the control group both in follow-ups ≥ 1 year and <1 year, as shown in Figure 4.

#### Uncorrected visual acuity (UCVA)

**Figure 5:**
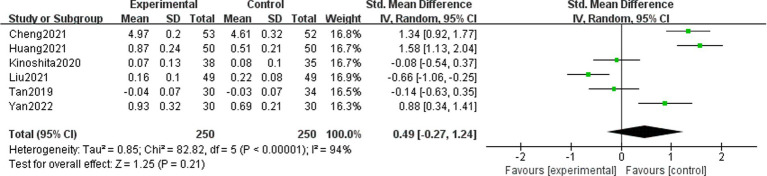
Forest plot of the meta-analysis for uncorrected visual acuity.

Six studies (12, 14–16, 20, 23) reported uncorrected visual acuity, with significant heterogeneity among the studies (*p* < 0.001, I^2^ = 94%). A random-effects model was applied after sensitivity analysis. The results indicated no statistically significant difference in UCVA between the two groups of children (SMD = 0.49, 95% CI = −0.27 to 1.24, *p* = 0.21), as shown in Figure 5. After excluding the study by Huang et al. in which the atropine concentration was 1%, the result remained statistically non-significant (SMD = 0.26, 95% CI = −0.49 to 1.02, *p* = 0.49).

#### Tear break-up time (TBUT)

**Figure 6:**
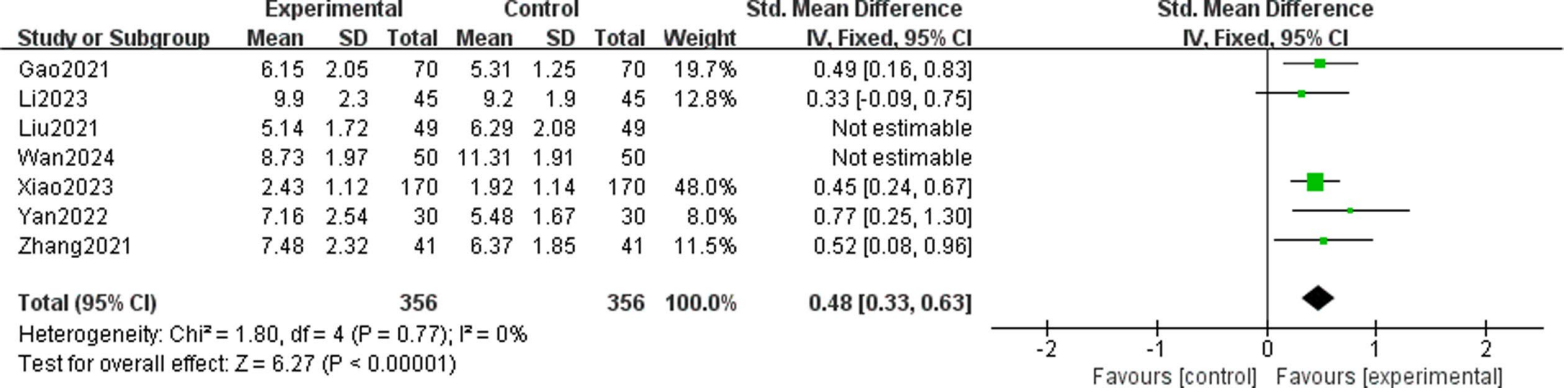
Forest plot of meta-analysis for tear break-up time.

Seven studies (10–13, 16, 18, 22) reported tear break-up time, with significant heterogeneity among the studies (*p* < 0.001, I^2^ = 92%). After sensitivity analysis excluding studies such as Liu (16) and Wang (10), no heterogeneity was observed among the remaining studies (*p* = 0.77, I^2^ = 0%), and a fixed-effects model was adopted. The meta-analysis results indicated that the tear break-up time in the intervention group was significantly longer than that in the control group (SMD = 0.48, 95% CI = 0.33–0.63, *p* < 0.001), as shown in Figure 6.

#### Tear film lipid layer thickness

**Figure 7:**
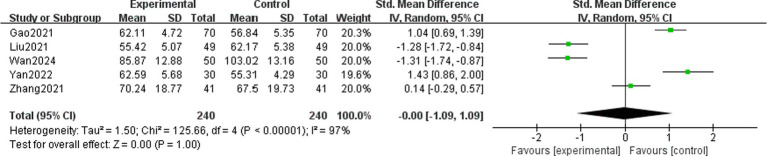
Forest plot of meta-analysis for the thickness of the tear film lipid layer.

Five studies (10, 12, 13, 16, 18) reported tear film lipid layer thickness, with significant heterogeneity among the studies (*p* < 0.001, I^2^ = 97%). A random-effects model was applied after sensitivity analysis. The meta-analysis results indicated no statistically significant difference in tear film lipid layer thickness between the two groups of children (SMD = −0.00, 95% CI = −1.09 to 1.09, *p* = 1.00), as shown in Figure 7.

#### Pupil diameter

**Figure 8:**
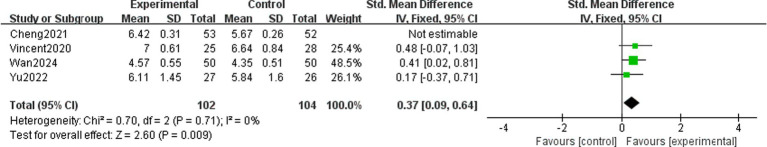
Forest plot of meta-analysis for pupil diameter.

Four studies (10, 14, 19, 21) reported pupil diameter, with significant heterogeneity among the studies (*p* < 0.001, I^2^ = 95%). After sensitivity analysis excluding the study by Cheng et al. (14), no heterogeneity was observed among the remaining studies (*p* = 0.71, I^2^ = 0%), and a fixed-effects model was adopted. The meta-analysis results indicated that the pupil diameter in the intervention group was larger than that in the control group (SMD = 0.37, 95% CI = 0.09–0.64, *p* < 0.001), as shown in Figure 8.

#### Accommodative amplitude

**Figure 9:**
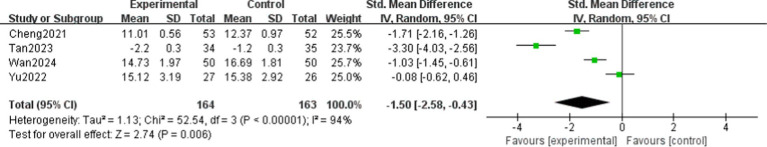
Forest plot of the meta-analysis on accommodative amplitude.

Four studies (10, 14, 21, 24) reported accommodative amplitude, with significant heterogeneity among studies (*p* < 0.001, I^2^ = 94%). A random-effects model was adopted after sensitivity analysis. The meta-analysis results indicated that the intervention group exhibited lower accommodative amplitude compared to the control group (SMD = −1.50, 95% CI = −2.58 to −0.43, *p* = 0.006), as shown in Figure 9.

#### Corneal curvature

**Figure 10:**
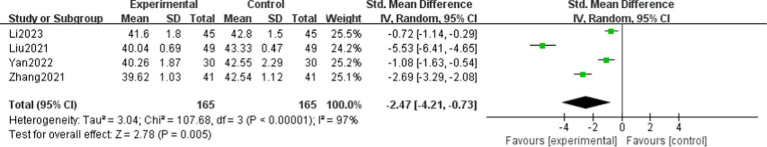
Forest plot of meta-analysis for corneal curvature.

Four studies (11–13, 16) reported corneal curvature, with significant heterogeneity among the studies (*p* < 0.001, I^2^ = 97%). A random-effects model was applied after sensitivity analysis. The meta-analysis results indicated that children in the intervention group had lower corneal curvature than those in the control group (SMD = −2.47, 95% CI = −4.21 to −0.73, *p* = 0.005), as shown in Figure 10.

### Publication bias assessment

**Figure 11:**
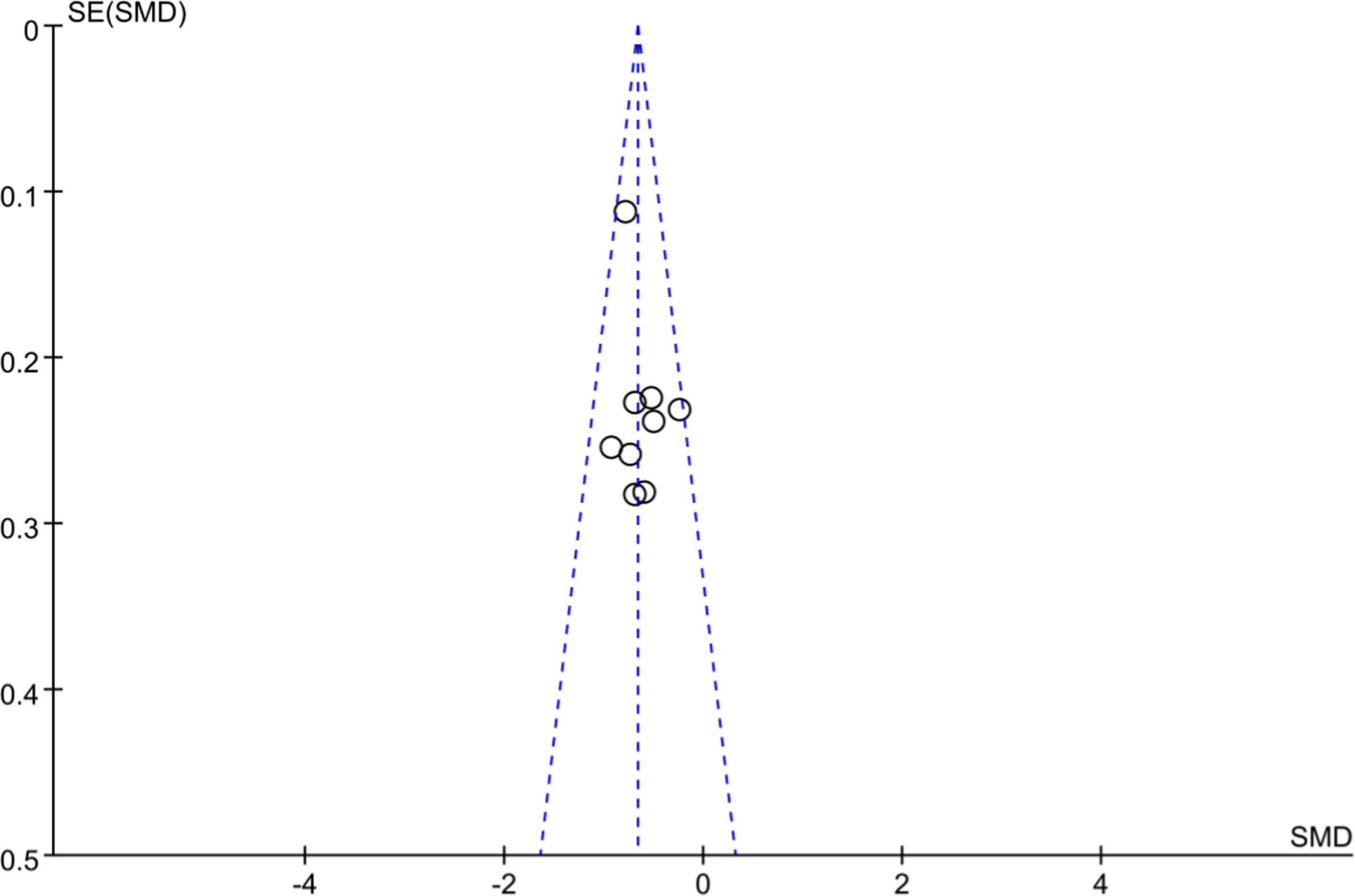
Funnel plot for publication bias assessment of axial elongation.

A publication bias assessment was conducted for indicators with seven or more included studies. Visual inspection of the funnel plot for axial elongation revealed a symmetrical and even distribution of studies without obvious gaps (Figure 11). The results of the Egger test further indicated no significant publication bias (Z = 1.06, *p* = 0.291).

### Summary of meta-analysis results

To enhance the reproducibility of the analysis, a summary of the Meta-analysis results is provided in Table 2.

**Table 2:** Summary of meta-analysis results.

No	Study	mean1	sd1	n1	mean2	sd2	n2	SMD [95%CI]
Axial elongation (mm)								
1	Hao, 2021 (26)	0.14	0.03	21	0.2	0.03	24	−1.96 [−2.69, −1.23]
2	Kinoshita, 2020 (23)	0.29	0.2	38	0.4	0.23	35	−0.51 [−0.98, −0.04]
3	Li, 2019 (17)	0.07	0.03	20	0.32	0.1	20	−3.32 [−4.30, −2.34]
4	Tan, 2019(20)	−0.05	0.05	30	−0.02	0.03	34	−0.73 [−1.24, −0.22]
5	Tan, 2023 (24)	0.17	0.19	34	0.35	0.2	35	−0.91 [−1.40, −0.42]
6	Vincent, 2020 (19)	−0.01	0.12	25	0.05	0.08	28	−0.59 [−1.14, −0.04]
7	Xiao, 2023 (22)	0.15	0.12	170	0.24	0.11	170	−0.78 [−1.00, −0.56]
8	Xu, 2023 (27)	0.31	0.23	42	0.43	0.22	40	−0.53 [−0.96, −0.10]
9	Yu, 2022 (21)	0.1	0.14	27	0.2	0.15	26	−0.68 [−1.23, −0.13]
10	Zhang, 2021 (13)	0.15	0.13	41	0.27	0.21	41	−0.68 [−1.13, −0.23]
11	Zhao, 2021 (25)	−0.01	0.05	39	0	0.03	36	−0.24 [−0.69, 0.21]
Overall								−0.24 [−0.69, 0.22]
Omitted-1								−0.71 [−0.85, −0.58]
Omitted-3								−0.71 [−0.84, −0.58]
Omitted-1, 3 (final adopted outcomes)								−0.66 [−0.80, −0.53]
Uncorrected visual acuity (logMAR)								
1	Cheng, 2021 (14)	4.97	0.2	53	4.61	0.32	52	1.34 [0.91, 1.77]
2	Huang, 2021 (15)	0.87	0.24	50	0.51	0.21	50	1.58 [1.13, 2.03]
3	Kinoshita, 2020 (23)	0.07	0.13	38	0.08	0.1	35	−0.08 [−0.53, 0.37]
4	Liu, 2021 (16)	0.16	0.1	49	0.22	0.08	49	−0.66 [−1.07, −0.25]
5	Tan, 2019(20)	−0.04	0.07	30	−0.03	0.07	34	−0.14 [−0.63, 0.35]
6	Yan, 2022 (12)	0.93	0.32	30	0.69	0.21	30	0.88 [0.35, 1.41]
Overall								0.49 [−0.27, 1.24]
Omitted-2 (final adopted outcomes)								0.26 [−0.49, 1.02]
Tear break-up time (seconds)								
1	Gao, 2021 (18)	6.15	2.05	70	5.31	1.25	70	0.49 [0.16, 0.82]
2	Li, 2023 (11)	9.9	2.3	45	9.2	1.9	45	0.33 [−0.08, 0.74]
3	Liu, 2021 (16)	5.14	1.72	49	6.29	2.08	49	−0.60 [−1.01, −0.19]
4	Wan, 2024 (10)	8.73	1.97	50	11.31	1.91	50	−1.32 [−1.75, −0.89]
5	Xiao, 2023 (22)	2.43	1.12	170	1.92	1.14	170	0.45 [0.23, 0.67]
6	Yan, 2022 (12)	7.16	2.54	30	5.48	1.67	30	0.77 [0.24, 1.30]
7	Zhang, 2021 (13)	7.48	2.32	41	6.37	1.85	41	0.52 [0.09, 0.95]
Overall								0.19 [0.06, 0.32]
Omitted-3								0.29 [0.15, 0.43]
Omitted-4								0.35 [0.21, 0.49]
Omitted-3, 4 (final adopted outcomes)								0.48 [0.33, 0.63]
Tear film lipid layer thickness (nm)								
1	Gao, 2021 (18)	62.11	4.72	70	56.84	5.35	70	1.04 [0.69, 1.39]
2	Liu, 2021 (16)	55.42	5.07	49	62.17	5.38	49	−1.28 [−1.71, −0.85]
3	Wang, 2024 (7)	85.87	12.88	50	103.02	13.16	50	−1.31 [−1.74, −0.88]
4	Yan, 2022 (12)	62.59	5.68	30	55.31	4.29	30	1.43 [0.86, 2.00]
5	Zhang, 2021 (13)	70.24	18.77	41	67.5	19.73	41	0.14 [−0.29, 0.57]
Overall (final adopted outcomes)								−0.00 [−1.09, 1.09]
Pupil diameter (mm)								
1	Cheng, 2021 (14)	6.42	0.31	53	5.67	0.26	52	2.60 [2.07, 3.13]
2	Vincent, 2020 (19)	7	0.61	25	6.64	0.84	28	0.48 [−0.07, 1.03]
3	Wang, 2024 (7)	4.57	0.55	50	4.35	0.51	50	0.41 [0.02, 0.80]
4	Yu, 2022 (21)	6.11	1.45	27	5.84	1.6	26	0.17 [−0.38, 0.72]
Overall								0.85 [0.61, 1.10]
Omitted-1 (final adopted outcomes)								0.37 [0.09, 0.64]
Accommodative amplitude (D)								
1	Cheng, 2021 (14)	11.01	0.56	53	12.37	0.97	52	−1.71 [−2.16, −1.26]
2	Tan, 2023 (24)	−2.2	0.3	34	−1.2	0.3	35	−3.30 [−4.03, −2.57]
3	Wang, 2024 (7)	14.73	1.97	50	16.69	1.81	50	−1.03 [−1.44, −0.62]
4	Yu, 2022 (21)	15.12	3.19	27	15.38	2.92	26	−0.08 [−0.61, 0.45]
Overall (final adopted outcomes)								−1.50 [−2.58, −0.43]
Corneal curvature (D)								
1	Li, 2023 (11)	41.6	1.8	45	42.8	1.5	45	−0.72 [−1.15, −0.29]
2	Liu, 2021 (16)	40.04	0.69	49	43.33	0.47	49	−5.53 [−6.41, −4.65]
3	Yan, 2022 (12)	40.26	1.87	30	42.55	2.29	30	−1.08 [−1.63, −0.53]
4	Zhang, 2021 (13)	39.62	1.03	41	42.54	1.12	41	−2.69 [−3.30, −2.08]
Overall (final adopted outcomes)								−2.47 [−4.21, −0.73]

### Summary of sensitivity analysis results

**Figure 12:**
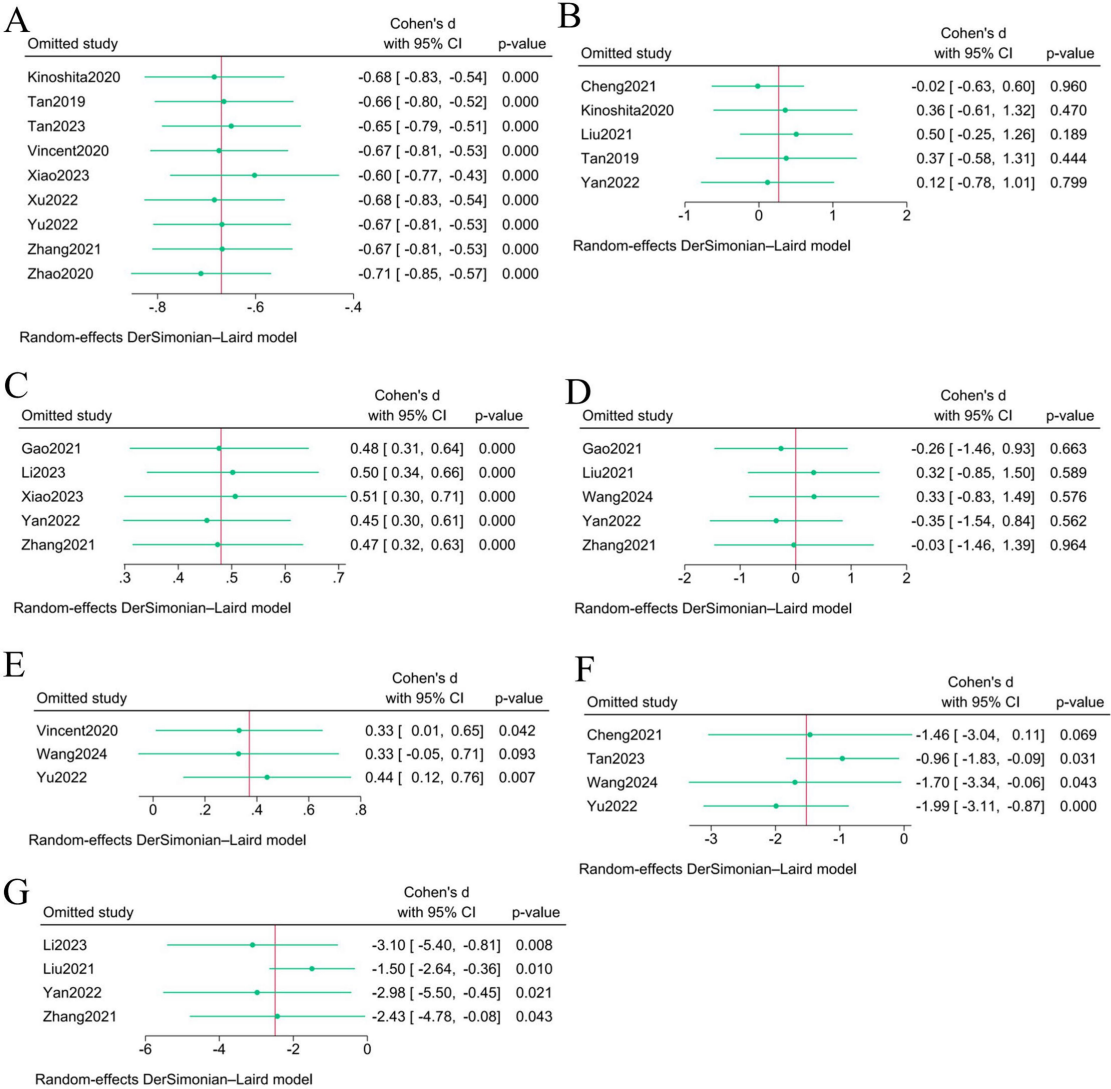
Forest plots of sensitivity analysis (leave-one-out method). **(A)** Axial elongation; **(B)** uncorrected visual acuity; **(C)** tear break-up time; **(D)** tear film lipid layer thickness; **(E)** pupil diameter; **(F)** accommodative amplitude; **(G)** corneal curvature.

To evaluate the robustness of the meta-analysis findings, leave-one-out sensitivity analyses were performed for all outcome indicators. The resulting forest plots are presented as a composite figure (Figure 12, panels A–G), corresponding to axial elongation, uncorrected visual acuity, tear break-up time, tear film lipid layer thickness, pupil diameter, accommodative amplitude, and corneal curvature, respectively, to visually demonstrate the impact of each individual study on the pooled effect sizes.

## Discussion

The earlier meta-analysis by Gao et al. (28), which included 5 studies involving 341 children, also reported an advantage of combination therapy in suppressing axial elongation, with a pooled effect showing a reduction in axial elongation of approximately 0.09 mm (WMD = −0.09 mm). Another earlier study by Wang S et al. (29), which included 4 studies with 267 children, found that the experimental group (orthokeratology + 0.01% atropine) had approximately 0.09 mm less axial elongation compared to the control group (WMD = −0.09, 95% CI: −0.15 to −0.03). These studies collectively suggest a consistent efficacy of the combined intervention in controlling axial elongation. Compared to these previous analyses, the present study includes a larger number of studies, a larger sample size, and encompasses a broader spectrum of outcome measures, including different atropine concentration regimens, variations in follow-up duration, and combined evaluation of axial length and refractive error. Furthermore, we have systematically incorporated recently published randomized controlled trials, which provides a more robust evidence base and enables a more detailed and representative comparison of the effects of different current clinical combination therapy strategies. Therefore, this study offers improvements over previous reviews in terms of both the comprehensiveness of the evidence and its clinical applicability.

Consistent with the observed advantages over previous studies, our meta-analysis demonstrated that the combination of orthokeratology and atropine therapy significantly reduces the axial elongation in children, a finding consistent with the results reported by Tsai et al. Compared with the effects of standalone orthokeratology treatment as described by Tsai et al. (30), the combined therapy exhibited superior efficacy in controlling axial elongation, suggesting a potential synergistic effect between the two interventions. The two modalities operate through complementary mechanisms. Orthokeratology primarily modifies the peripheral refractive power distribution of the cornea, thereby reducing peripheral retinal hyperopic defocus, while low-dose atropine suppresses axial elongation by modulating retinal dopaminergic signaling and remodeling the scleral extracellular matrix (31). Furthermore, the combined treatment may produce an additive effect. Recent studies (4) indicate that the peripheral defocus signals generated by orthokeratology enhance retinal sensitivity to atropine, whereas atropine may optimize the optical effects of orthokeratology by modulating choroidal thickness. This bidirectional potentiation is reflected at the molecular level as a synergistic improvement in retinal dopamine levels and scleral collagen fiber alignment. Notably, subgroup analysis revealed that follow-up duration (≥1 year or <1 year) had no significant impact on treatment efficacy, a finding that differs from previous studies (32). We speculate that this may be due to the relatively concentrated follow-up periods in the included studies (mostly 1–2 years), making it difficult to detect long-term differences. Additionally, the age-dependent variation in myopia progression rates among children (33), along with the observed decline in treatment efficacy beyond the first 12 months of optical interventions (34), may have obscured the influence of time factors.

The results of this meta-analysis on uncorrected visual acuity (UCVA) and ocular surface health indicators provide important clinical insights. Regarding UCVA, the combination therapy group showed no significant difference compared to the monotherapy orthokeratology group, a finding consistent with the results reported by Guo et al. (35). Possible explanations include: both treatment modalities effectively correct central corneal refractive power, achieving comparable daytime visual outcomes; or the low-concentration atropine (0.01%) exerts only a limited pupil-dilating effect, with no significant impact on visual quality (36). In terms of ocular surface health, the combination therapy group demonstrated a longer tear break-up time (TBUT), a noteworthy finding that is supported by recent evidence demonstrating better preservation of tear film stability with the combined therapy (37). Contrary to previous assumptions that atropine may exacerbate dry eye (23), our results suggest that low-concentration atropine combined with orthokeratology therapy may have a protective effect on the ocular surface. The anti-inflammatory properties of atropine mitigate orthokeratology-related ocular surface microinflammation, while standardized orthokeratology wear improves meibomian gland function. Notably, no significant difference was observed in tear film lipid layer thickness between the two groups, which appears contradictory to the improvement in TBUT. This may be because tear film stability is influenced by multiple factors, and lipid layer thickness is not the sole determinant (38). This finding highlights the need for a comprehensive assessment of multiple indicators when evaluating ocular surface health in clinical practice.

This meta-analysis on pupil diameter, accommodative amplitude, and corneal curvature reveals the multidimensional effects of combined orthokeratology and atropine therapy on the pediatric visual system. Regarding pupil diameter, the combined treatment group exhibited significant enlargement, which aligns precisely with the pharmacological mechanism of atropine. In terms of accommodative amplitude, the combined treatment group demonstrated a notable decline in accommodative amplitude, a finding that is fully consistent with the pharmacological mechanism of atropine and aligns with the results reported by Yu et al. (21). Further analysis indicates that this effect primarily stems from atropine’s blockade of M3 receptors in the ciliary muscle. Of particular note, the degree of accommodative amplitude reduction was comparable to that observed in previous studies on 0.05% atropine monotherapy (39, 40), suggesting that orthokeratology-induced reshaping of the anterior corneal surface may potentially influence accommodative demand. A recent systematic review and meta-analysis further confirmed that atropine slows myopia progression in a dose-dependent manner, with greater efficacy observed in East Asian populations (41). This finding has implications for clinical practice, recommending regular monitoring of accommodative amplitude in children undergoing combined therapy, with supplementary vision training if necessary. Changes in corneal curvature primarily reflect the therapeutic effects of orthokeratology, mirroring results reported in standalone orthokeratology studies (13). This confirms that atropine use in combined therapy does not significantly alter the corneal reshaping efficacy of orthokeratology. It should be noted that although some studies used millimeters as the unit of measurement for axial length, significant differences remained among the studies in terms of measuring devices and timepoints. Furthermore, different units of measurement and instruments were used for other outcomes (such as corneal curvature and tear film parameters). To avoid the inability to pool studies due to unit inconsistency, this study employed SMD as the effect size to ensure comparability across different studies and the robustness of the analysis.

This meta-analysis has several limitations: First, there was significant heterogeneity in intervention protocols among the included studies, with variations in orthokeratology lens designs and follow-up durations, which may affect the accuracy of the results. Second, methodological limitations include insufficient standardization of measurements. The timing of corneal curvature measurements varied across studies (some taken 24 h after discontinuing orthokeratology lenses, others after 48 h), potentially leading to measurement bias. Third, there are limitations in the characteristics of the study population. The average age range of the included children was relatively narrow, with a lack of specific analysis for younger children, while treatment responses may vary across different age groups. Fourth, the included studies reported limited outcome measures, and for some outcome indicators, there was insufficient information to conduct effective subgroup analyses to explore the sources of heterogeneity. Fifth, the included studies did not report adverse effects, making it impossible to evaluate the safety of orthokeratology combined with atropine treatment. Finally, there is a lack of long-term efficacy data. The maximum follow-up duration in the included studies was only 2 years, making it impossible to evaluate the long-term effects of combination therapy, particularly the cumulative impact on accommodative amplitude and rebound phenomena after treatment cessation.

In conclusion, compared with orthokeratology monotherapy, the combination of orthokeratology and atropine therapy demonstrates reduced axial elongation, increased tear break-up time, and reduced corneal curvature in children with myopia. However, no significant advantage was observed in terms of uncorrected visual acuity or tear film lipid layer thickness. Meanwhile, indicators of increased pupil diameter and reduced accommodative amplitude reflect the side effects associated with atropine combination therapy. Nevertheless, due to the high heterogeneity in some outcome measures, the lack of long-term follow-up data, and age-specific analyses, future large-scale, high-quality, and standardized studies are still needed to focus on optimizing treatment parameters, evaluating long-term safety, and developing withdrawal strategies. Such efforts will provide more robust evidence for precise myopia control in children.

## Data Availability

The original contributions presented in the study are included in the article/supplementary material, further inquiries can be directed to the corresponding author.
